# Whole Genome Sequencing Reveals Multiple Linked Genetic Variants on Canine Chromosome 12 Associated with Risk for Symmetrical Lupoid Onychodystrophy (SLO) in the Bearded Collie

**DOI:** 10.3390/genes12081265

**Published:** 2021-08-19

**Authors:** Liza C. Gershony, Janelle M. Belanger, Marjo K. Hytönen, Hannes Lohi, Anita M. Oberbauer

**Affiliations:** 1 Department on Animal Science, University of California, Davis, CA 95616, USA; lcgershony@ucdavis.edu (L.C.G.); jmbelanger@ucdavis.edu (J.M.B.); 2 Department of Medical and Clinical Genetics, University of Helsinki, 00014 Helsinki, Finland; marjo.hytonen@helsinki.fi (M.K.H.); hannes.lohi@helsinki.fi (H.L.); 3 Department of Veterinary Biosciences, University of Helsinki, 00014 Helsinki, Finland; 4 Folkhälsan Research Center, 00290 Helsinki, Finland

**Keywords:** SLO, onychodystrophy, onychomadesis, dogs, autoimmune, DLA, MHC, WGS, genomics, imputation

## Abstract

In dogs, symmetrical lupoid onychodystrophy (SLO) results in nail loss and an abnormal regrowth of the claws. In Bearded Collies, an autoimmune nature has been suggested because certain dog leukocyte antigen (DLA) class II haplotypes are associated with the condition. A genome-wide association study of the Bearded Collie revealed two regions of association that conferred risk for disease: one on canine chromosome (CFA) 12 that encompasses the DLA genes, and one on CFA17. Case-control association was employed on whole genome sequencing data to uncover putative causative variants in SLO within the CFA12 and CFA17 associated regions. Genotype imputation was then employed to refine variants of interest. Although no SLO-associated protein-coding variants were identified on CFA17, multiple variants, many with predicted damaging effects, were identified within potential candidate genes on CFA12. Furthermore, many potentially damaging alleles were fully correlated with the presence of DLA class II risk haplotypes for SLO, suggesting that the variants may reflect DLA class II haplotype association with disease or vice versa. Strong linkage disequilibrium in the region precluded the ability to isolate and assess the individual or combined effect of variants on disease development. Nonetheless, all were predictive of risk for SLO and, with judicious assessment, their application in selective breeding may prove useful to reduce the incidence of SLO in the breed.

## 1. Introduction

Symmetrical lupoid onychodystrophy (SLO; OMIA 001989-9615) is a painful condition afflicting the nails of dogs. It is characterized by inflammation of the nail bed with secondary bacterial infection that causes the claws to slough off. Regrowth of nails is often abnormal, resulting in deformed and brittle claws in otherwise healthy dogs [1,2,3,4]. While uncommon in the overall dog population, a relatively higher prevalence of SLO has been described in Bearded Collies [4,5], German Shepherds [6], and Gordon and English Setters [7].

Disease etiology remains unclear, although an autoimmune component has been suggested and supported by recent studies [3,8,9]. For example, certain major histocompatibility complex (MHC; or dog leukocyte antigen, DLA) class II haplotypes are more frequently seen in Bearded Collies and Gordon Setters that have SLO [8,9,10], and are thus considered risk haplotypes for the disease. Genome-wide association studies (GWAS) for SLO in both Bearded Collies and Gordon Setters have indicated a region of association on canine chromosome (CFA) 12 that includes the DLA class II genes [3,11], further supporting the involvement of the DLA genes in SLO disease development. 

However, stretches of linkage disequilibrium (LD) ranging from 0.4 to 3.2 megabases (Mb) long are known to exist within dog breeds [12,13]. While this genomic structure provides an advantage for studying genetic association with phenotypes in dogs, where association mapping requires fewer markers than in humans [13], fine mapping of causative variants can be more challenging. As in humans, the MHC region of the dog genome exhibits particularly long stretches of strong LD [14], and numerous genes are found in that genomic region of CFA12. It is therefore possible that the DLA association identified is a result of LD rather than true causation. For example, in Bearded Collies, the strongest association observed in the GWAS was seen with a single nucleotide polymorphism (SNP) located in the intronic region of a tenascin gene (*TNXB*), and that *TNXB* GWAS SNP was fully correlated with the presence of DLA class II risk haplotypes for SLO [11]. 

In Bearded Collies, a second region of association was also identified on CFA17. Moreover, a stronger association of the CFA17 region was noted when only Bearded Collies carrying DLA class II risk haplotypes were included in the GWAS. Despite its clear association with SLO, no obvious additive effect of the CFA17 locus on the risk for disease conferred by the CFA12 locus was noted. A potential candidate gene (regenerating family member 3 alpha, *REG3A*) was identified adjacent to the associated region on CFA17. The REG3A protein functions to modulate epidermal repair after skin injury by regulating keratinocyte proliferation and differentiation [15]. A dog’s nail beds are under constant mechanical stress and periods of intense training are thought to contribute to SLO disease onset [3], thus making this gene a good candidate for SLO disease risk.

Many potential candidate genes, beyond the DLA, exist in the CFA12 region indicated by the GWAS and a relatively small number are found in the CFA17 region. The purpose of this study was to explore whole genome sequencing (WGS) data from SLO-affected and healthy Bearded Collies in order to identify putative causative variants within the two GWAS-associated regions on CFA12 and CFA17. To refine the large number of WGS variants observed in the regions of interest, genotype imputation, which has been shown to accurately predict WGS genotypes based on data from high-density SNP arrays [16,17,18], was also employed.

## 2. Materials and Methods

### 2.1. Samples

Whole blood samples were obtained from SLO and healthy Bearded Collies. Dogs included as SLO cases had been diagnosed by a veterinarian based on clinical signs, including brittle nails, inflamed nail bed, nail bleeding, single or multiple nail loss with subsequent abnormal nail growth, and exclusion of other potential causes of nail loss; three SLO dogs also had a biopsy of the nail beds showing signs consistent with SLO. Dogs selected as healthy controls were clinically healthy and at least 10 years old, with no history of nail abnormalities or immune-related disorders, diagnosed or suspected. DNA was extracted from blood samples, as previously described [19], and quantified using a Nanodrop^®^ spectrophotometer and Qubit^®^ fluorometer.

### 2.2. WGS and Variant Calling

DNA aliquots from 26 Bearded Collies (7 with SLO, 8 healthy controls above 10 years, and 11 Bearded Collies that were free from SLO and sequenced for other studies) were submitted to Novogene (Novogene, Beijing, China) for WGS. The SLO dogs (3 females, 4 males) and healthy control dogs (2 females, 6 males) were unrelated up to the grandparent level, except for one SLO and one control dog that shared a grandsire. The SLO dogs included the three cases with nail bed biopsies. Age at SLO diagnosis ranged from 20 months to 7 years (average age, 4.5 years), consistent with the literature [4,20]. Healthy control dogs were from 11 to 15 years old (median age 12.5 years). The Illumina HiSeq X™ Ten platform was used to sequence a PCR-free library with fragment sizes of approximately 350 bp to obtain 150 bp paired-end reads and an average of 15× coverage. Raw sequence data files were processed according to the Broad Institute’s Genome Analysis Tool Kit (GATK) best practices workflow for small germline variants [21]. Briefly, FastQC [22] was used to check the quality of the raw reads, which were then subjected to quality-based trimming with Trimmomatic version 0.39 [23]. Trimmed reads were aligned to the CanFam 3.1 reference genome available on Ensembl [24] using BWA-MEM version 0.7.16a [25]. Some samples had been run on more than one sequencing lane so that each of those dogs contained 2–3 mapped files. For those samples, Samtools version 1.9 [26] was used to sort and index each mapped file and Picard-tools version 2.23.4 [27] was used to merge the files into a single combined mapped file per sample. In preparation for variant calling, Picard-tools was also used to mark duplicate reads and GATK version 4.2.0.0 was used for base recalibration. GATK’s HaplotypeCaller was then used to call variants in each individual sample, followed by joint variant calling across all samples. Variant files were then annotated with SnpEff version 5.0e [28] using the CanFam3.1 reference genome, and Ensembl annotation version 104 was used to add information about the location of each variant relative to the genes and the effect of those variants on resulting proteins (i.e., synonymous, missense, frameshift, etc.). 

### 2.3. Variant Filtering for Case-Control Association on 7 SLO and 8 Healthy Controls

Given that haplotype blocks significantly associated with SLO had been identified in CFA12 between 1.2 Mb and 6.5 Mb [11], variants called within the first 7.5 Mb of CFA12 were extracted from the main WGS dataset for the 7 SLO and 8 healthy controls for further analysis; similarly, variants located between 44 and 47 Mb of CFA17 were also extracted. These ranges allowed for approximately 1 Mb flanking region on each side of the GWAS SLO-associated regions. Individual call rates within the regions of association were calculated using PLINK version 1.9 [29]; samples with less than 90% call rate were excluded from further analysis. Variant files were filtered using VCFtools version 0.1.17 [30], keeping variants called in at least 70% of dogs (i.e., 11 of 15 dogs), having a minor allele frequency (MAF) greater than 5%, and supported by at least 3 reads. Additionally, variant files for controls were filtered in PLINK to remove any variants that deviated from the Hardy–Weinberg equilibrium (HWE) at *p*-value < 0.00005. Case-control *p*-values were calculated for each of the remaining variants using SnpSift, a tool that uses 2 × 2 contingency tables to count the number of non-reference genotypes in the case and control groups and uses the Fisher exact test to calculate allelic *p*-values for each variant [31,32]. Variants with an allelic case-control *p*-value < 0.05 and SnpEff-predicted high or moderate impact (Supplemental Table S1) were then selected for further investigation. 

### 2.4. Imputation

**Figure 1:**
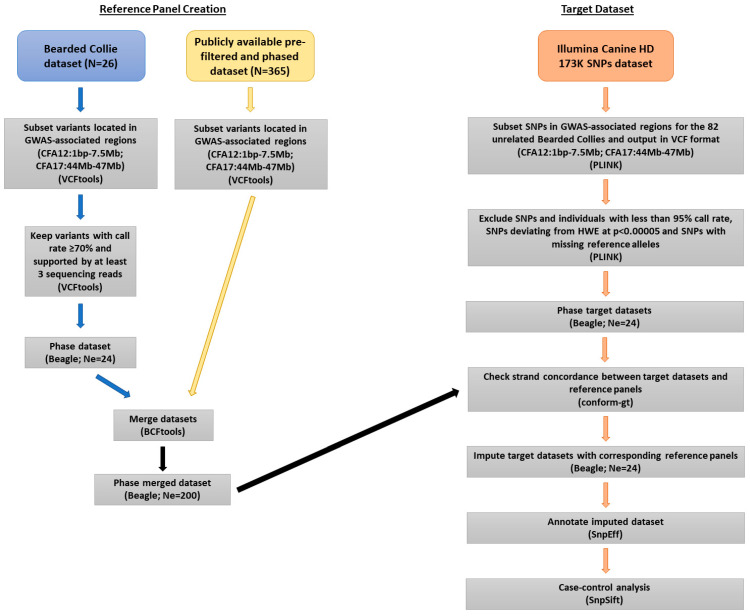
Flowchart illustrating dataset processing for imputation. Software and relevant parameters used are indicated at each step. The publicly available dataset required no additional filtering and phasing prior to merging. CFA, canine chromosome; N, number of individuals; Ne, effective population size; SNP, single nucleotide polymorphism; HWE, Hardy–Weinberg equilibrium; bp, base pairs; Mb, megabases.

Genotype imputation was employed in an effort to refine the variants of interest in the associated regions of CFA12 and CFA17. A reference panel for imputation was generated from two datasets: our laboratory’s variant dataset obtained from WGS of 26 Bearded Collies and a publicly available phased dataset obtained from WGS of 365 canids [16]. Both datasets were processed according to GATK’s best practices workflow. Variants located within the first 7.5 Mb of CFA12 were extracted from each of the datasets using VCFtools; the same was performed for variants located between 44 and 47 Mb of CFA17 (Figure 1). Individual call rates calculated by PLINK for the 26 Bearded Collies prior to filtering ranged from 93.3% to 96.3% for CFA12 and from 94.9% to 97.3% for CFA17; thus, no samples were excluded. To reduce the number of unreliable variants and genotype calls while preserving as much genetic variability intrinsic to Bearded Collies as possible, filtering was applied to the Bearded Collie datasets using VCFtools so that variants with less than a 70% call rate across all individuals and genotypes supported by fewer than 3 reads were discarded. No MAF or HWE filtering was performed when creating the reference panel for imputation, given that disease variants are expected to be present at low frequencies in the population and any deviation from HWE could be an indication of genetic disease association, which is why HWE filtering should only be applied to known controls in case-control studies [33]. Genotype phasing was performed using Beagle version 5.1 [34] and default parameters [35], but adjusting the effective population size (Ne) to 24. Since the datasets consisted only of Bearded Collies, and a 2015 UK study reported an Ne of 23.91 for the breed [36], an effective population size of 24 was deemed appropriate. Bcftools [26] version 1.2 was used to merge the CFA12 and CFA17 Bearded Collie subset datasets to the corresponding subsets of the publicly available dataset. Beagle 5.1 was then used to phase the genotypes in the merged datasets using default parameters, but this time adjusting Ne to 200, as described in a previously published imputation study of a multibreed dataset [17]. Additionally, the publicly available average CFA12 and CFA17 genetic maps [37] were used as input for phasing. Finally, the datasets to be used as reference panels for imputation contained 391 samples and 111,740 variants for CFA12, and 391 samples and 20,854 variants for CFA17. Among the 391 samples, 236 were dogs belonging to 77 breeds, 26 of which were Bearded Collies; 107 were village dogs; and 28 were wolves [16]. The target dataset was generated using genotype data from 82 unrelated Bearded Collies (30 SLO and 52 healthy) included in a previous GWAS [11]. Genotypes for the Illumina Canine HD BeadChip SNPs located within the first 7.5 Mb of CFA12 and SNPs located between 44 and 47 Mb of CFA17 were extracted and used to impute up to the 111,740 and 20,854 WGS variants, respectively. The locations of the Illumina SNPs were based on the CanFam3.1 reference genome available on Ensembl. PLINK version 1.9 [29] was used to subset the genotype dataset and convert the files into VCF format. SNPs and individuals with less than 95% call rate were removed from the dataset as well as SNPs deviating from HWE at *p* < 0.00005 in the controls. Illumina SNPs with missing non-reference alleles after VCF conversion were also removed (N = 32 for CFA12 and N = 11 for CFA17). After filtering, the CFA12 target dataset contained 524 SNPs with a 98.7% call rate across the 82 unrelated dogs; the CFA17 contained 210 SNPs with a 99.8% call rate across samples. Beagle 5.1 was used to phase the genotypes without imputing missing calls and an Ne of 24 was used for phasing. Conform-gt [38] was then used to check for strand concordance between the target datasets and the CFA12 and CFA17 reference panels, and variants found to be strand concordant (N = 516 and N = 182, respectively) were used for imputation. Beagle 5.1 was used to impute genotypes [34,39] using the newly generated CFA12 and CFA17 reference panels and an Ne of 24. The imputed VCF files were annotated using SnpEff [28], and SnpSift [31] was used for a case-control analysis of each variant; the case-control analysis included the 82 dogs from the GWAS [11]. Variants were then filtered based on allelic case-control *p*-value < 0.05 using SnpSift and compared to the variants of interest identified in the WGS data. Novel variants of interest (i.e., variants not matching a previously known dbSNP record) were checked through a manual inspection of mapped reads using the Integrative Genomics Viewer (IGV) [40] to confirm variant calls prior to further investigation. Confirmed variants of interest were then checked for variant effect using the Protein Variation Effect Analyzer (Provean) online tool [41], which accepts any species for analysis and has been validated to predict the effect of amino acid substitutions and inframe insertions or deletions (indels) on the biological function of a protein [42]. Genomic Evolutionary Rate Profiling (GERP) scores [43], based on the alignment of 90 mammalian species, were obtained from Ensembl for each variant and used to assess the conservation of the particular amino acid across mammals. The VassarStats online tool [44] was used to calculate the odds ratio (OR) and the two-tailed Fisher’s exact *p*-values of the variants of interest supported by the 82 unrelated Bearded Collies through 2 × 2 contingency tables. Calculations were based on the number of cases and controls that carried a particular genotype versus the number of cases and controls not carrying that genotype. Statistically significant observations were considered at *p*-value < 0.05. A VCF file containing the 11 variants of interest was recoded into binary files in PLINK, and pairwise LD statistics were then calculated and plotted using the genetic data handling package “gaston” version 1.5.7 [45] implemented in R [46]. 

### 2.5. Imputation Accuracy

Imputation accuracy was assessed using 24 of the 26 WGS of Bearded Collies that had also been run on the Illumina Canine HD Beadchip. Target datasets were created for estimating the imputation accuracy of the CFA12 and CFA17 reference panels using the same methods described above but including genotype data from the 24 WGS Bearded Collies only. The same imputation parameters used for imputing the 82 unrelated Bearded Collies were applied to the 24 WGS dogs. Following imputation as described above, Bcftools was used to compare each dog’s imputed genotype to their WGS genotype called by GATK. Imputation accuracy was estimated by determining the proportion of correct genotype calls per dog (i.e., number of matching genotypes divided by the total number of variants). 

### 2.6. TNXB Sequencing

Based upon the previous GWAS association [11], where the most significant SNP was intronic to *TNXB*, 73 Bearded Collies from our database (35 SLO, 38 healthy controls) were selected and genotyped for three missense variants in *TNXB* identified by WGS, which were predicted to be deleterious by Provean [42]. Thirty-two of these 73 dogs (20 SLO and 12 controls) had been included in the previous GWAS analysis; other dogs that had been assessed in the GWAS could not be used for the *TNXB* sequencing because their DNA was no longer available. Primers flanking each of the three SNPs were designed using Primer 3 [47] (TNXB_925-F:CTTGTATTGGACCACGAAGGAG, TNXB_925-R:TTCTATGCTCCAAGCTCCAAAG; TNXB_337-F:GGAGGCCCATGAAGCACA, TNXB_337-R:CTGCCCATCCCCATTCTTGT; TNXB_360-F:GGCCTCTTTCTCTCACGTCC, TNXB_360-R:CCCGTCTTCATGCCCTGG). A standard 33-cycle polymerase chain reaction (PCR) protocol was used to amplify each product using Promega GoTaq^®^ Flexi DNA Polymerase (Promega, Madison, WI, USA) in a 25 µL reaction. A 62 °C annealing temperature was used for the TNXB_925 primer set and 60 °C for the TNXB_337 and TNXB_360 primer sets. Amplicon size was checked through gel electrophoresis, using 5 µL of the PCR product run on a 1% agarose gel. Exosap-IT™ express (Thermo Fisher Scientific, Waltham, MA, USA) was used to purify the PCR products, and sequencing was performed by capillary electrophoresis on an ABI 3730 DNA analyzer (Applied Biosystems, Foster City, CA, USA). Sequences were read with FinchTV v.1.4 (Geospiza, CO, USA) and aligned against the CanFam3.1 reference sequence using CLC viewer v.7.8.1 (QIAGEN Bioinformatics, Aarhus, Denmark) for genotyping.

## 3. Results

### 3.1. WGS

**Figure 2:**
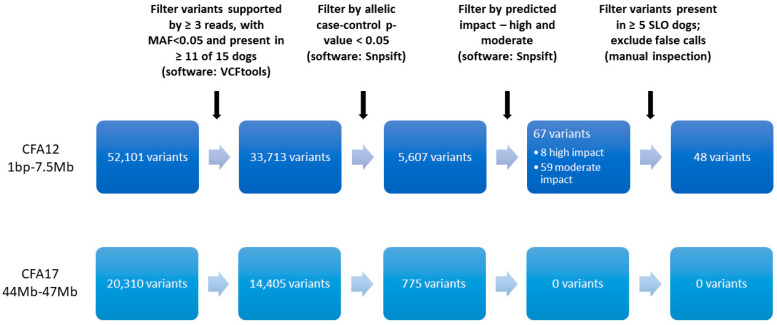
Filtering process of whole genome sequence variants identified within the GWAS regions of association on canine chromosomes (CFA) 12 and 17 for further exploration of their association with symmetrical lupoid onychodystrophy (SLO) in Bearded Collies. Chromosome locations are based on the CanFam3.1 reference genome. Software used in each step indicated in parenthesis.

The mapping of WGS data from the 26 Bearded Collies to the CanFam3.1 reference genome resulted in an average genome-wide coverage of 15.6× per sample, with individual dogs ranging from 11 to 20× coverage. After extracting variant data for the 15 Bearded Collies (7 SLO, 8 healthy controls) for SLO analysis, 52,101 variants were present within the first 7.5 Mb of CFA12 and 20,310 variants were present between 44 and 47 Mb of CFA17. The individual call rate for the 15 Bearded Collies ranged from 93.4% to 96.5% on CFA12 and from 95.5% to 97.6% on CFA17 prior to filtering. Of the variants called within the CFA12 and CFA17 associated regions, 33,713 and 14,405 variants, respectively, were supported by at least 3 reads, called in at least 70% of the dogs, and presented with an MAF greater than 5%. Individual call rate after filtering ranged from 95.2% to 99% for CFA12 and from 96.0% to 98.6% for CFA17. Variants were then filtered based on an allelic case-control *p*-value < 0.05, leaving 5,607 variants for analysis on CFA12 (Figure 2). More importantly, all variants in the associated region on CFA17 were eliminated. Of the 5607 variants on CFA12, 8 were predicted to have a high impact and 59 were predicted to have a moderate impact, as defined by SnpEff; 49 of those having moderate or high predicted impact were present in at least 5 of the WGS SLO cases. One variant was excluded as a false call based on the manual inspection of mapped reads. 

### 3.2. Variants from Imputed Dataset

Genotype imputation of WGS variants was employed on the 82 unrelated Bearded Collies included in the previous GWAS in an effort to refine the list of variants for further exploration. Imputation accuracy, estimated as the percentage of correctly imputed genotypes in 24 Bearded Collies, averaged 91.7% for SNPs (range 81.3–98.3%) and 87.6% for insertions or deletions (i.e., indels; range 78.5–97.8%) on CFA12, and 86.8% for SNPs (range 75.1–99.4%) and 84.6% for indels (range 73.0–98.9%) on CFA17, similar to what has previously been achieved with a multi-breed reference panel [16,17,18].

Of the 48 variants identified in the CFA12 WGS data, 43 were present in at least 25 of 30 unrelated cases and remained significantly associated with SLO in the imputed dataset (i.e., case-control allelic *p*-value < 0.05; Supplementary Table S2). The effect of 32 of those 43 variants on the resulting protein was predicted to be neutral by Provean, thus eliminating them from further analysis. The remaining 11 variants of interest consisted of 7 missense mutations, 3 frameshift variants, and 1 splice acceptor variant (Table 1). The missense variants were predicted to be deleterious by Provean, including the three found in *TNXB*. Though Provean is not currently able to ascribe a consequence to frameshift and splice acceptor variants, they can have damaging consequences on the resulting protein: frameshift variants can change the reading frame beginning at the site of insertion/deletion and can lead to several amino acids being changed in the protein or even a premature stop codon [48], whereas splice acceptor variants can impair proper gene splicing [49].

**Figure 3:**
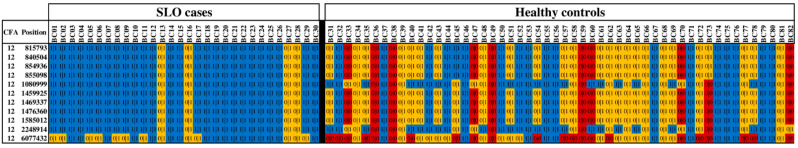
Genotypes for the 11 variants of interest on canine chromosome 12 in 82 unrelated Bearded Collies (30 SLO and 52 healthy controls). Dogs were sorted by SLO status; the homozygous reference genotypes (red) are notably absent in the SLO dogs, which predominantly carry homozygous non-reference (blue) genotypes. Heterozygous genotypes are colored in yellow. Variant locations are based on the CanFam3.1 reference genome. CFA—canine chromosome; SLO—symmetrical lupoid onychodystrophy.

The homozygous reference genotype was completely absent from the SLO group at all 11 variants of interest (Figure 3), and almost all SLO dogs (87%) presented a homozygous non-reference genotype at those locations, with the exception of the last variant (rs852291453) in which only 17 cases were homozygous non-reference and 13 were heterozygous.

### 3.3. TNXB Sequencing

Given that the top genome-wide significant SNP from the GWAS was intronic to *TNXB,* a gene that encodes an extracellular matrix protein that is ubiquitously expressed in the skin, and provided that the majority of SLO dogs was homozygous for the non-reference allele at all three missense variants predicted to have deleterious effects on the resulting protein, highest priority was given to investigating these variants in additional dogs.

Of the variants observed in *TNXB*, 11 were missense variants with an allelic case-control association *p*-value < 0.05. Three of those (rs22185869, rs8493203, and rs853176058) were predicted by Provean to have deleterious consequences in all known transcripts. The variants resulted in the substitution of a proline to a leucine, a proline to an arginine, and an aspartic acid to a glycine, respectively. The first two variants displayed negative GERP scores in Ensembl, indicating a high variability of amino acids in those positions across mammalian species; however, the third variant (rs853176058) had the highest positive GERP score of all variants of interest in Table 1, indicating high conservation across species and suggesting that alterations at this site could impact protein function. 

All seven WGS SLO dogs were homozygous for the non-reference allele at all three *TNXB* SNPs, whereas two controls were homozygous and three were heterozygous for the missense substitutions. Genotyping of 73 additional Bearded Collies (35 SLO and 38 healthy controls) confirmed the association of the deleterious SNPs with SLO (Table 2). For an additional evaluation of the quality of imputation, genotype concordance in the imputed data was also checked for the three missense variants predicted to be deleterious in *TNXB*. Thirty-two of the 73 Bearded Collies sequenced for *TNXB* were also included in the GWAS and their imputed genotypes fully matched the genotypes obtained through sequencing. 

Two DLA class II risk haplotypes have been previously associated with increased risk for SLO in Bearded Collies [8,11]. Dogs that were homozygous for the non-reference allele at all three *TNXB* SNPs also carried two DLA class II risk haplotypes for SLO (i.e., either homozygous for one of the risk haplotypes or carrying one of each of the two DLA class II risk haplotypes), whereas dogs that were heterozygous at all three SNPs carried one risk and one non-risk DLA class II haplotype. Dogs that were homozygous reference at the SNPs had no DLA class II risk haplotypes. More specifically, there was direct concordance with the presence of the *TNXB* variants and that of the DLA class II haplotypes identified as conferring risk.

### 3.4. Additional Variants on CFA12

The variants identified in the CFA12 associated region affected 8 genes, one of which (ENSCAFG00000041540) is defined as a pseudogene in Ensembl. In addition to *TNXB*, four genes (lymphotoxin B, *LTB*; G protein signaling modulator 3, *GPSM3*; major histocompatibility complex, class II, DQ beta 2, *HLA-DQB2;* and FYVE, RhoGEF and PH domain containing 2, *FGD2*) could be considered potential candidate genes given their role in the immune system. The variants in *LTB* and *HLA-DQB2* had the lowest ORs, with the non-reference and potentially deleterious allele being present in a homozygous state in many control dogs. The second lowest OR was observed for the *FGD2* variant, which was the only variant of interest where the majority of SLO dogs were not homozygous for the variant. The *GPSM3* variant, while promoting a frameshift at the very beginning of the coding sequence that causes the resulting protein to be significantly shorter than the wildtype, only affected one of three known transcripts. Although not clearly associated with the immune system, of the remaining variants, those in the transcription factor 19 gene (*TCF19*) affected all known transcripts and resulted in a premature stop codon and a smaller resulting protein, which could indicate significant impairment of function. 

**Figure 4:**
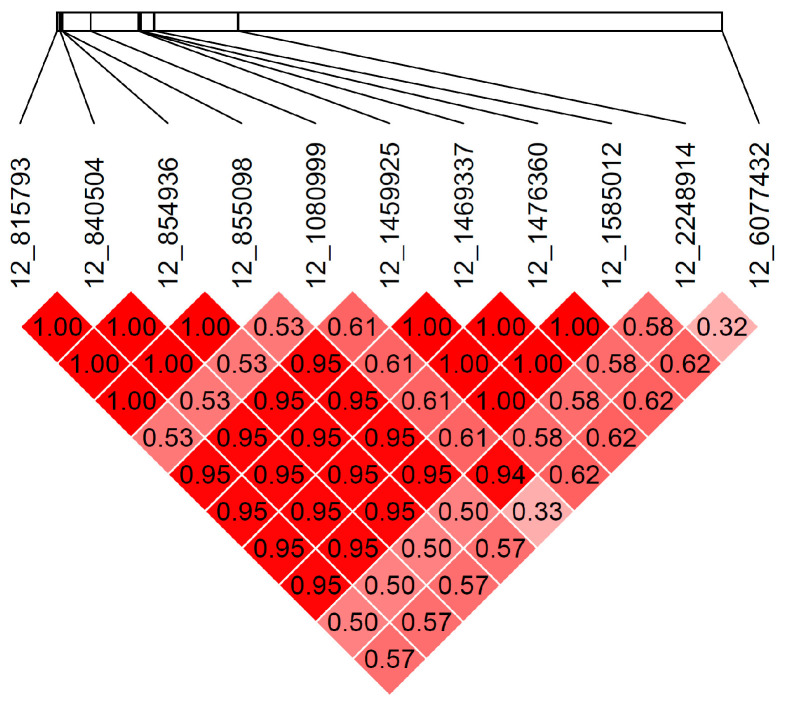
Chromosome 12 linkage disequilibrium matrix showing pairwise r-squared values for the imputed genotypes of 82 unrelated Bearded Collies at 11 variants of interest. Variants are labeled by chromosome and location, as defined by the CanFam3.1 reference genome. Image generated using the R package gaston. The intensity of red corresponds to the r-squared value, with deeper red indicating greater r-squared values.

Further sequencing of the *TCF19*, *LTB*, and *GPSM3* variants in more dogs was considered. However, pairwise LD calculations demonstrated moderate to strong linkage disequilibrium throughout the entire region (Figure 3 and Figure 4), most especially between variants in *TCF19*, *LTB*, *GPSM3,* and *TNXB*, indicating that the observed deleterious variants would all follow the same pattern of direct linkage with the DLA class II risk haplotypes, as seen with the *TNXB* sequencing. Therefore, additional sequencing of these genes would fail to resolve whether they or the DLA class II risk haplotypes were causal or correlated. 

### 3.5. CFA17

No variants of interest (with high or moderate impact) were identified on CFA17, according to the criteria used to select variants for further investigation. Given that a potential candidate gene was identified adjacent to the CFA17 region of association, variants called within that gene were reviewed separately. Forty-two variants were called in *REG3A*: one in the five prime untranslated region (UTR), four in the three prime UTR, 36 intronic, and one in the coding region of the gene, which was synonymous and predicted to have low impact on the resulting protein. None of the variants in either UTR or intron 1 of *REG3A* presented with an allelic case-control *p*-value < 0.05 among the WGS dogs or the 82 unrelated dogs. Therefore, none of these variants were investigated further.

## 4. Discussion

The investigation of WGS data in an effort to define causal variants in SLO in Bearded Collies revealed several potentially damaging variants within the risk-associated region of CFA12 that were homozygous in the majority of SLO dogs. The variants on CFA12 affected 8 protein-coding genes, one defined as a pseudogene with no known orthologues in Ensembl. The highest ORs associated with SLO disease status were observed for three missense variants in *TNXB*, one of which is reported to be highly conserved across mammalian species, and a frameshift variant in the adjacent *GPSM3* gene. *TNXB* encodes the extracellular matrix protein tenascin-X found extensively in the skin, including the dermal papillae of the human nail matrix and hyponychium [50], where it organizes and maintains connective tissue structure [51]. A SNP in human *TNXB* has been associated with systemic lupus erythematosus [52], although its role in autoimmunity remains unclear. Combined with the top GWAS SNP for SLO being intronic to *TNXB* [11], the gene was considered a potential candidate for SLO susceptibility. The three missense variants in *TNXB* were predicted to be deleterious and were present at significantly greater frequency in SLO dogs compared to controls. Two of the three missense variants, including that for the highly conserved position, result in the substitution of a non-polar to polar amino acid (proline to arginine and aspartic acid to glycine), which could affect the 3-dimensional structure of the resulting protein. 

Missense variants in *TNXB* have recently been implicated in skin hyperextensibility in a mixed-breed dog, generating a phenotype reminiscent of Ehlers–Danlos syndrome. Those two missense variants (c.2012G > A and c.2900G > A) were extremely rare in the overall dog population and only seen in the heterozygous state [53]. Though indels or truncating mutations are more commonly associated with Ehlers–Danlos syndrome, missense variants in human tenascin-X have also been reported in connection with the hyperextensibility of the skin and joints [54]. Although none of the Bearded Collies included in this study carried either of those mutations mentioned above, and none displayed any systemic skin or joint abnormalities, the finding does support a role for *TNXB* in canine integument lending further support for a potential role of *TNXB* in SLO. 

In addition to *TNXB*, five other protein-coding genes harbored variants considered as possible candidate variants in SLO disease development: *TCF19*, *LTB*, *GPSM3*, *HLA-DQB2,* and *FGD2*. Out of these, the *HLA-DQB2* variant could be considered the strongest candidate for SLO, given that the variant is in exon 2 of the gene, which encodes the binding site of the DLA molecule produced [55]. MHC molecules are instrumental to central tolerance and, through their binding sites, participate in thymic T-cell development leading to deletion of potentially autoreactive T cells [56]. Certain MHC class II haplotypes are thought to contribute to autoimmunity by failing to eliminate autoreactive T cells, allowing them to escape thymic selection [56,57]. Sequencing of the exon 2 of each of the three polymorphic DLA class II genes (major histocompatibility complex, class II, DR beta 1, *DLA-DRB1*; major histocompatibility complex, class II, DQ alpha 1, *DLA-DQA1*; and *HLA-DQB2*) is used to determine three-locus DLA class II haplotypes [8,58], and the variant of interest identified in this study is shared by four DLA class II haplotypes seen in Bearded Collies, including the two very similar haplotypes that were associated with increased risk for SLO [8]. This would explain the lower OR seen with this variant and why risk for disease is better captured by the presence of one or two three-locus DLA class II risk haplotypes for SLO, which remains more informative of disease risk in Bearded Collies than the individual *HLA-DQB2* variant (rs851008370).

Among the remaining variants of interest, the one in *LTB* (rs852946032) might be considered the second strongest candidate variant given its *LTB* gene function. *LTB* encodes the lymphotoxin β protein, which is a member of the tumor necrosis factor superfamily and critical to immune system development [59]. The gene is known to be important in regulating innate and adaptive immune responses [60] and has been found to be up-regulated in a group of human inflammatory diseases, thus suggesting that *LTB* may play a role in perpetuating inflammation [61]. Moreover, LTB deficiency has been shown to prevent autoimmunity in non-obese diabetic mice, allergic encephalomyelitis, and experimental murine colitis models [59]. However, the *LTB* variant identified in this study also had a low OR, and a large number of control dogs was found to be homozygous for the non-reference and potentially deleterious allele. Canine *LTB* has two transcripts, one resulting in a much smaller protein than the other. The observed variant associated with SLO in the Bearded Collie causes a glycine at position 52 of the protein to be substituted by glutamic acid; this corresponds to the first fourth of the larger transcript and the last third of the smaller transcript. Although the variant affects both transcripts, Provean predicted it to be neutral in the larger transcript and deleterious in the smaller one. The C-terminal end of the protein, which is likely to be exposed on the cell surface and possibly involved in receptor binding, is the most conserved region of the protein [62], thus supporting Provean predictions. While it is possible that there is a transcript dosage effect influencing SLO expression, with the predicted deleterious transcript reducing the functionality of the LTB protein, it is also possible that the larger transcript is able to compensate for the smaller transcript and that the variant observed in *LTB* would not be sufficient to induce disease in the Bearded Collies.

The last gene with a clear involvement in immune function is *FGD2*, which is expressed in leukocytes, B cells, macrophages, and dendritic cells, and plays a role in immune system signaling and vesicle trafficking [63]. While its function in the immune system could make it a candidate gene for SLO, the associated variant identified in this gene also had one of the lowest ORs, with cases and controls presenting similar proportions of homozygous non-reference and heterozygous genotypes. While it may still be contributing to SLO susceptibility in combination with other variants, there is no clear evidence of this from our findings. 

Frameshift variants can have severe consequences on the resulting protein [48], especially when they occur towards the beginning of the coding sequence. This is the case for the variant in *GPSM3* (rs851873877)*,* which causes a frameshift starting at the third amino acid of the protein, introducing a premature stop codon and thereby terminating the protein sequence at only 30 amino acids. As expected, the variant also presented with the strongest OR and LD along with the *TNXB* variants, given their physical proximity on the chromosome. Human GWAS studies have pointed to an inverse correlation between *GPSM3* and autoimmune diseases, demonstrating that polymorphisms in this gene were associated with the decreased incidence of immune-mediated diseases such as rheumatoid arthritis and systemic lupus erythematosus (SLE) [64]. While clearly causing significant changes to the resulting protein, the identified variant only affects one of the known canine *GPSM3* transcripts and is intronic to the other two. It is therefore possible that the other transcripts compensate for the affected one. Combined with the strong LD observed with other variants identified in this study, it is unclear whether this variant could have a role in SLO disease development or is simply linked to the true causative variant(s). 

The two frameshift variants in *TCF19* (novel variants: p.Gln114fs and p.Trp164fs)*,* leading to significant amino acid changes in the first fourth of the protein as well as a premature stop codon, were strongly associated with SLO and also in strong LD with the *TNXB*, *LTB,* and *GPSM3* variants. The *TCF19* gene encodes a transcription factor involved in regulating cell proliferation and differentiation and has been implicated in type 1 diabetes where it appears to act as a regulator of β-cell mass in the pancreas [65,66]. Despite its association with another organ-specific autoimmune disorder, there is no other evidence in the literature to connect this gene’s function to SLO development. Finally, although the variants in *C12H6orf15* and *ENSCAFG00000041540* (defined as a pseudogene in Ensembl) had a significant association with SLO, there is little known about these genes and how they may predispose to SLO. Furthermore, the high LD evident between these variants and variants located in stronger candidate genes that have a clear potential involvement in disease development, reduce the likelihood of their contribution to SLO risk.

Despite a definite association between CFA17 and SLO [11], no variants of interest were identified throughout the associated region. A separate investigation of variants within a potential candidate gene adjacent to the associated region also failed to reveal any variants significantly segregating with disease status. The region of association on CFA17 is relatively gene-poor, with few regulatory motifs. It is possible that the region may harbor regulatory elements that could influence the expression of the adjacent *REG3A* gene, but no such variants were obvious.

While conferring an advantage to association mapping in the dog [13], long stretches of LD can make fine mapping of true causative variants more challenging, as exemplified by the present study. A significant challenge in the search for WGS variants that increase risk susceptibility for SLO is that the entire region of association on CFA12 is in strong LD. Genotype imputation in a larger number of unrelated cases and controls allowed five of the variants identified in WGS to be discarded from further consideration. As previously reported, Bearded Collies carry two types of DLA class II haplotypes that are strongly associated with SLO disease development [8,11]. While the involvement of *TNXB* seems plausible, given its ubiquitous expression in the skin and the presence of a non-reference allele at each of the *TNXB* variant locations, the variants were fully linked to the presence of DLA class II risk haplotypes for SLO. In fact, the three *TNXB* variants were also in strong LD, with almost all of the other variants of interest identified, making it difficult to determine which variant, or combination of variants, may play a role in SLO susceptibility in Bearded Collies. After evaluating the role of each of the variants in the candidate genes and assessing their potential involvement based upon protein function, a logical conclusion is that the risk susceptibility observed likely reflects the DLA class II haplotypes. The presence of variants in the candidate genes are inextricably linked to the presence of the DLA class II risk haplotypes, creating one very large risk haplotype encompassing many genes. Given the genetic structure of the region, successfully isolating the different variants to assess their effects on SLO disease development may not be possible even if additional dogs were sequenced at the whole genome level. Since the GWAS-associated region on CFA12 was similar for Bearded Collies and Gordon Setters, sequencing of SLO and healthy dogs from other SLO-affected breeds such as the Gordon Setter may help clarify which of the variants on CFA12 are associated with disease and which ones are simply in LD with the causative variants, assuming the genetic cause of SLO is the same across dog breeds and the variants are not in LD in different breeds. It should also be noted that the methodology employed in the current study focused on variants with a high or moderate SnpEff-predicted impact. Variants in introns and untranslated regions of genes as well as intergenic variants, which are considered by SnpEff to have only modifier effects, were excluded from analysis by our filtering process. However, such variants may play a role in disease development through the regulation of gene expression, and further exploration of such variants within the associated region could reveal additional associations with disease. Alternatively, although an invasive procedure, a biopsy and the subsequent gene expression profiling of the nail beds of SLO affected and unaffected dogs might shed light on which of these genes are truly associated with the condition. 

Despite the limitations, the present study was able to refine the associated region on CFA12, identifying several genetic variants in potential candidate genes that have predicted damaging effects and are strongly associated with SLO disease expression in the Bearded Collies. In terms of biological function, the DLA class II genes may still be the most probable causal genes in SLO, although a combination of the DLA class II haplotypes with one or more of the variants identified in this study cannot be excluded. Given that the potentially damaging variants and DLA class II risk haplotypes for SLO are quite abundant in the Bearded Collie population, strong selection against these alleles could result in a significant loss of genetic diversity to the breed. Nonetheless, the variants and DLA class II risk haplotypes are strongly predictive of SLO status in this breed and, if used judiciously, their application to inform breeding decisions may prove helpful to reduce the incidence of SLO in the breed population. 

## Figures and Tables

**Table 1:** Variants of interest selected for further exploration on canine chromosome (CFA) 12. Odds ratio (OR) calculated for the homozygous non-reference genotype in 82 unrelated Bearded Collies (30 SLO, 52 controls). Locations are based on the CanFam3.1 reference genome.

CFA12 Position	Variant Rs Number *	Gene	All Transcripts Affected?	Variant Type	GERP Conservation Score ^§^	SLO (N = 30)		Controls (N = 52)		OR (95% CI)	*p*-Value ^¶^
						HO ALT	HET	HO ALT	HET		
815793	rs851051888	C12H6orf15	Yes	Missense	0.16	26	4	18	25	12.3 (3.7–40.7)	0.000007
840504	rs851625125	ENSCAFG00000041540	Yes	Splice acceptor	−0.15	26	4	18	25	12.3 (3.7–40.7)	0.000007
854936	Novel	TCF19	Yes	Frameshift	NA	26	4	18	25	12.3 (3.7–40.7)	0.000007
855098	Novel	TCF19	Yes	Frameshift	NA	26	4	18	25	12.3 (3.7–40.7)	0.000007
1080999	rs852946032	LTB	Yes	Missense	0.16	26	4	32	15	4.1 (1.2–13.4)	0.022579
1459925	rs22185869	TNXB	Yes	Missense	−0.86	26	4	17	25	13.4 (4.0–44.5)	0.000002
1469337	rs8493203	TNXB	Yes	Missense	−4.06	26	4	17	25	13.4 (4.0–44.5)	0.000002
1476360	rs853176058	TNXB	Yes	Missense	2.66	26	4	17	25	13.4 (4.0–44.5)	0.000002
1585012	rs851873877	GPSM3	No	Frameshift	0.16	26	4	17	25	13.4 (4.0–44.5)	0.000002
2248914	rs851008370	HLA-DQB2	No	Missense	0.16	26	4	32	15	4.1 (1.2–13.4)	0.022579
6077432	rs852291453	FGD2	No	Missense	0.41	17	13	12	18	4.4 (1.7–11.5)	0.003677

* Previously reported variant identification number obtained from Ensembl; ^§^ Genomic Evolutionary Rate Profiling score based on the alignment of 90 mammalian species; ^¶^ Two-tailed Fisher’s Exact *p*-value; CI—confidence interval; SLO—symmetrical lupoid onychodystrophy; HO ALT—homozygous non-reference genotype; HET—heterozygous genotype.

**Table 2:** Genotypes at each of the three missense variants predicted to be deleterious in *TNXB* for Bearded Collies and the type of DLA class II haplotypes (i.e., risk haplotype or non-risk haplotype for SLO) associated with those *TNXB* variant combinations. Variant locations are based on the CanFam3.1 reference genome.

Genotype at Each Variant Location			SLO (N = 42)	Controls (N = 46)	OR (95% CI)	*p*-Value ^¶^	DLA Class II Risk Haplotypes ^1^
12:1459925	12:1469337	12:1476360					
GG	GG	TT	1	8	0.12 (0.01–0.97)	0.03139	No risk haplotypes
GA	GC	TC	5	23	0.14 (0.05–0.41)	0.00018	1 risk haplotype
AA	CC	CC	36	15	12.4 (4.29–35.85)	4.1 × 10^−7^	2 risk haplotypes

^1^ Risk haplotypes defined in [8]; ^¶^ Two-tailed Fisher’s Exact *p*-value; OR—odds ratio; CI—confidence interval; SLO—symmetrical lupoid onychodystrophy.

## Data Availability

All pertinent data analyzed in this study are included in the published article and its supplementary information file (Supplementary Table).

## Supplementary Materials

The following are available online at https://www.mdpi.com/article/10.3390/genes12081265/s1, Table S1: Types of variants with moderate or high impact according to SnpEff; Table S2: Variants of interest and corresponding Provean predictions. Location and affected genes are based on the CanFam3.1 reference genome.
