# Supplementary material for: Whole Genome Sequencing Reveals Multiple Linked Genetic Variants on Canine Chromosome 12 Associated with Risk for Symmetrical Lupoid Onychodystrophy (SLO) in the Bearded Collie
Source: Genes (Basel). 2021 Aug 19;12(8):1265. doi: 10.3390/genes12081265 (PMC8394396; doi:10.3390/genes12081265)
Supplement: Supplementary file 1 [file genes-12-01265-s001.zip › genes-1326550-supplementary/Supplementary File 1.pdf]

Table S1. Types of variants with moderate or high impact according to SnpEff [32].

Moderate Impact Variants	High Impact Variants
inframe insertion	exon loss variant
disruptive inframe insertion	duplication
inframe deletion	inversion
disruptive inframe deletion	frameshift
missense	feature ablation
splice region variant	gene fusion
3 prime UTR truncation + exon loss	bidirectional gene fusion
5 prime UTR truncation + exon loss variant	rearranged at DNA level
Sequence feature + exon loss variant	protein-protein contact
	structural interaction variant
	rare amino acid variant
	splice acceptor variant
	splice donor variant
	stop lost
	start lost
	stop gained

Source: Cingolani, P. SnpEff & SnpSift Documentation. Available online: http://pcingola.github.io/SnpEff/ss_casecontrol/ (accessed on 08/10/2021)
