# Supplementary material for: Whole Genome Sequencing Reveals Multiple Linked Genetic Variants on Canine Chromosome 12 Associated with Risk for Symmetrical Lupoid Onychodystrophy (SLO) in the Bearded Collie
Source: Genes (Basel). 2021 Aug 19;12(8):1265. doi: 10.3390/genes12081265 (PMC8394396; doi:10.3390/genes12081265)
Supplement: Supplementary file 1 [file genes-12-01265-s001.zip › genes-1326550-supplementary/Supplementary File 2.pdf]

Table S2. Variants of interest and corresponding Provean [42, 43] predictions. Location and affected genes are based on the CanFam3.1 reference genome available on Ensembl [24].

CFA	POS	Provean Prediction	Variant Type	Gene Symbol or Ensembl ID
12	663014	Neutral	missense variant	DDR1
12	682971	Neutral	missense variant	VARS2
12	815793	Deleterious	missense variant	C12H6orf15
12	815806	Neutral	missense variant	C12H6orf15
12	840504	NA	splice acceptor variant & intron variant	ENSCAFG00000041540
12	854936	NA	frameshift variant	TCF19
12	855098	NA	frameshift variant	TCF19
12	857756	Neutral	missense variant	TCF19
12	857757	Neutral	missense variant	TCF19
12	858526	Neutral	missense variant	TCF19
12	894038	Neutral	missense variant	DLA88
12	983519	Neutral	missense variant	DLA-64
12	1080999	Neutral for 201; Deleterious for transcript 202	missense variant	LTB
12	1105607	Neutral	missense variant	AIF1
12	1193544	Neutral	missense variant	LY6G6D
12	1201059	Neutral	missense variant	LY6G6C
12	1268748	Neutral	missense variant	VARS1
12	1279238	Neutral	missense variant	ENSCAFG00000035349
12	1279343	Neutral	disruptive inframe deletion	ENSCAFG00000035349
12	1300624	Neutral	missense variant	ENSCAFG00000000641
12	1417675	Neutral	missense variant	SKIV2L
12	1437707	Neutral	missense variant	ENSCAFG00000000701
12	1438500	Neutral	missense variant	ENSCAFG00000000701
12	1438623	Neutral	missense variant	ENSCAFG00000000701
12	1440096	Neutral	missense variant	ENSCAFG00000000701
12	1459925	Deleterious	missense variant	TNXB
12	1461286	Neutral	missense variant	TNXB
12	1469050	Neutral	missense variant	TNXB
12	1469337	Deleterious	missense variant	TNXB
12	1470758	Neutral	missense variant	TNXB
12	1470833	Neutral	missense variant	TNXB
12	1476360	Deleterious	missense variant	TNXB
12	1476467	Neutral	missense variant	TNXB
12	1476809	Neutral	missense variant	TNXB
12	1489066	Neutral	missense variant	TNXB
12	1500451	Neutral	missense variant	TNXB
12	1526455	Neutral	missense variant	FKBPL
12	1547611	Neutral	disruptive inframe deletion	PRRT1
12	1574485	Neutral	conservative inframe deletion	AGER
12	1585012	NA	frameshift variant	GPSM3
12	2225122	Neutral	missense variant	DLA-DQA1
12	2248914	Deleterious	missense variant	HLA-DQB2
12	6077432	Deleterious	missense variant	FGD2

CFA – canine chromosome; NA – not applicable – Provean is not able to assess variant type; POS – position of variant based on the CanFam3.1 reference genome

[42]Choi, Y.; Sims, G.E.; Murphy, S.; Miller, J.R.; Chan, A.P. Predicting the Functional Effect of Amino Acid Substitutions and Indels. *PLOS ONE* **2012**, *7*, e46688, doi:10.1371/journal.pone.0046688.

[43]Choi, Y.; Chan, A.P. PROVEAN web server: a tool to predict the functional effect of amino acid substitutions and indels. *Bioinformatics* **2015**, *31*, 2745-2747, doi:10.1093/bioinformatics/btv195

[24]Howe, K.L.; Achuthan, P.; Allen, J.; Allen, J.; Alvarez-Jarreta, J.; Amode, M.R.; Armean, I.M.; Azov, A.G.; Bennett, R.; Bhai, J.; et al. Ensembl 2021. Nucleic Acids Research 2020, 49, D884-D891, doi:10.1093/nar/gkaa942
